# Radial Undersampling-Based Interpolation Scheme for Multislice CSMRI Reconstruction Techniques

**DOI:** 10.1155/2021/6638588

**Published:** 2021-04-12

**Authors:** Maria Murad, Abdul Jalil, Muhammad Bilal, Shahid Ikram, Ahmad Ali, Baber Khan, Khizer Mehmood

**Affiliations:** ^1^Department of Electrical Engineering, International Islamic University Islamabad, Islamabad 44000, Pakistan; ^2^Department of Software Engineering, Bahria University Islamabad, Islamabad 44000, Pakistan

## Abstract

Magnetic Resonance Imaging (MRI) is an important yet slow medical imaging modality. Compressed sensing (CS) theory has enabled to accelerate the MRI acquisition process using some nonlinear reconstruction techniques from even 10% of the Nyquist samples. In recent years, interpolated compressed sensing (iCS) has further reduced the scan time, as compared to CS, by exploiting the strong interslice correlation of multislice MRI. In this paper, an improved efficient interpolated compressed sensing (EiCS) technique is proposed using radial undersampling schemes. The proposed efficient interpolation technique uses three consecutive slices to estimate the missing samples of the central target slice from its two neighboring slices. Seven different evaluation metrics are used to analyze the performance of the proposed technique such as structural similarity index measurement (SSIM), feature similarity index measurement (FSIM), mean square error (MSE), peak signal to noise ratio (PSNR), correlation (CORR), sharpness index (SI), and perceptual image quality evaluator (PIQE) and compared with the latest interpolation techniques. The simulation results show that the proposed EiCS technique has improved image quality and performance using both golden angle and uniform angle radial sampling patterns, with an even lower sampling ratio and maximum information content and using a more practical sampling scheme.

## 1. Introduction

The Shannon-Nyquist theorem [1] states that a signal can only be reconstructed from its *k*-space data if it has a sufficient number of samples, which are at minimum twice the maximum frequency in that signal. But if in an imaging modality the signal acquisition time is highly dependent on the number of samples it has to acquire, like MRI, then the Nyquist theorem becomes a real bottleneck. The slow image acquisition process of MRI makes it inapplicable in emergency and accidental cases. Secondly, this time-consuming process also causes a claustrophobic feeling, particularly in pediatric patients, and it is difficult for them to be motionless and in the breath-held state for that long [2]. Although there are many different approaches in MRI acquisition that can accelerate this process like parallel imaging [3–6], CS can efficiently reduce the average samples [7–9] and scan time to up to 10 times by just increasing the computational complexity. The 10% random samples acquired for CS reconstruction [8, 10–12] do not show aliasing artifacts rather they have a noise-like effect [13–16]. MRI has got benefit from CS because it fulfills their three fundamental constraints which are (i) sparsity, (ii) incoherence, and (iii) nonlinear signal recovery [10]. Unlike a normal MRI scan, where the acquired *k*-space data only require inverse Fourier transform, compressed sensing MRI (CSMRI) needs some nonlinear reconstruction techniques [10, 11] which are an additional computational overhead. But this computational load is just a postacquisition process and does not bother a patient by compelling it to stay with the MRI machine. CS has been implemented using both Cartesian and non-Cartesian undersampling schemes [12, 17, 18].

**Figure 1:**
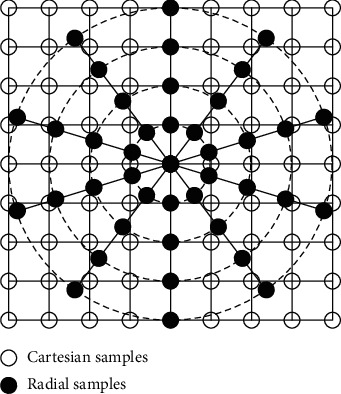
Gridding radial samples on the Cartesian grid [21].

Non-Cartesian sampling in *k*-space has appeared in many medical imaging modalities like MRI. Radial sampling has evolved since the beginning of MRI, with the limitation that its nonuniformly spaced samples of the spatial frequency domain are to be projected on uniformly spaced samples in the image domain [19].  Figure 1 shows some polar radial samples which are to be projected on a Cartesian grid. The value of each Cartesian sample is to be determined from the samples of the adjacent radial samples through gridding reconstruction [20] which uses Nonuniform FFT (NUFFT) [19] and a Density Compensation Function (DCF). The DCF helps to mitigate the artifacts caused by the overrepresentation of some spatial frequencies in non-Cartesian acquisitions. Similarly, for converting uniformly sampled Cartesian image data into nonuniform *k*-space data, inverse gridding is used [19].

Image reconstruction using radial undersampling has been rapidly evolved as it allows reduced scan time with increased spatial resolution. The iterative reconstruction of CS forms an undersampled radially encoded MRI dataset that is helpful for artifact-free images [17, 18, 21–23]. These artifacts are directly related to the number of samples available for reconstruction. Thus, if we first estimate the missing samples in the highly undersampled radially encoded multislice MRI datasets, before CS reconstruction, one can get an alias-free reconstructed image from just a fraction of the total samples.

**Figure 2:**
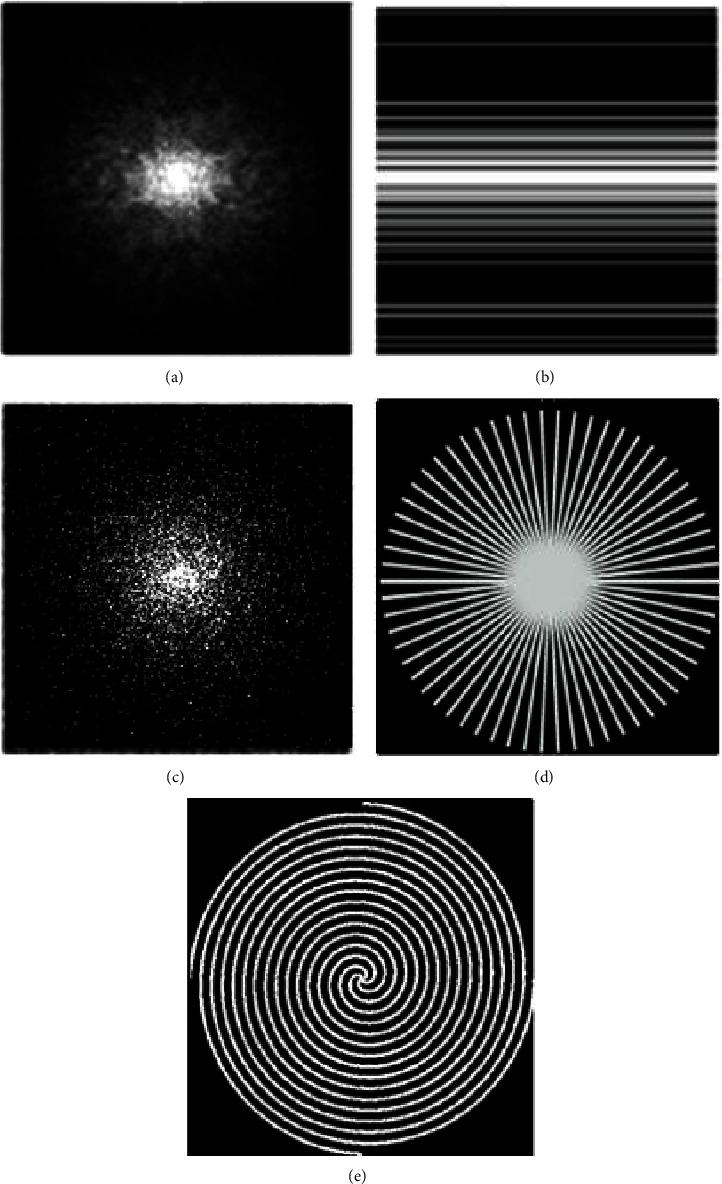
(a) Original *k*-space and undersampled *k*-space slices using a (b) 1D-VRDU, (c) 2D-VRDU, (d) radial, and (e) spiral mask.

A single multislice MRI scan acquires hundreds of slices. Therefore, their consecutive slices have a very strong interslice correlation, because of having very narrow interslice gaps [24]. In recent years, many researchers have exploited this correlation of multislice MRI for further reduction of the scan time, through interpolation. This new concept is termed as interpolated compressed sensing (iCS) in the literature [24, 25]. Through iCS, the average sampling ratio of CSMRI has been reduced even beyond the CS limit. Interpolated compressed sensing mainly works in three steps: (i) undersampling the multislice MRI data, (ii) interpolation, and (iii) CS reconstruction. For the first step, the desired undersampling is done using a much lower undersampling ratio compared to CS. In iCS undersampling, some of the CS samples are missed intentionally to reduce the average sampling ratio and scan time. The random undersampling of iCS can be accomplished using one of the many undersampling approaches like Cartesian, radial, spiral, and their combinations [17, 26–28].  Figure 2 shows the original *k*-space of multislice MRI and some different undersampling approaches that can be used in CSMRI. The second step of iCS approximates the missed samples of the highly undersampled slices from the samples of their neighboring slices [24, 25, 29, 30]. The aim of this interpolation [24, 25, 30–32] is to get CS slices. Finally, in the third step, CS reconstruction techniques [10, 17, 32–37] are applied on the interpolated slices to get reconstructed multislice MRI datasets.

The concept of iCS has been introduced by Pang et al. [24, 25] in 2012. His interpolation technique has later been explored by Datta and Deka [31, 32], but their undersampling approaches do not produce clinically acceptable results by causing information loss in most (67%) of their reconstructed slices [38]. Secondly, their interpolation techniques are computationally inefficient with redundant Fourier steps. Although their results look visually better, the information content is not indigenous due to their nonuniform and a biased undersampling scheme [32]. Pang and Zhang's [25] work is on 2D-VRDU where 1D-VRDU is explored by Datta and Deka [31, 32]. Their works on both 1D- and 2D-VRDU sampling schemes have inaccuracy along with complex computational steps of convolution, matrix division, Fourier, and inverse Fourier transforms. Afterwards, Datta and Deka [39–42] have further explored iCSMRI with different reconstruction strategies and interpolation approaches along with reduced computational cost compared to their initial work [31, 32]. But their work has reduced neither the average undersampling ratios nor the undersampling strategies, rather they have increased the average sampling ratio [39].

In a recent work [38], the authors proposed a new and fast interpolation technique (FiCS) based on a 2D-VRDU sampling scheme. Their outcome shows more clinically acceptable results with less partial volume loss and lower average sampling ratio and by using a computational efficient interpolation technique. The interpolation approach of FiCS is a simple two-step process utilizing two consecutive slices to estimate the missing samples of each target slice (*T* slice) from its corresponding left slice (*L* slice). FiCS has reduced the average undersampling ratio to 5%, compared to the previous iCS techniques which have at minimum 9% average samples. The results of FiCS also show improvement in terms of information content and image quality with even half of the sampling ratio compared to their previous interpolation techniques. Moreover, the interpolation technique of FiCS is very computational efficient with just a two-step process having only set addition and difference operations. But the basic limitation of FiCS is that their undersampling strategy does not apply to current clinical scanners and their images lack sharpness.

The recent trends of CSMRI can be classified as techniques dedicated to improved reconstruction techniques [43–45] and parallel CSMRI approaches [4, 5, 40, 42, 46, 47]. In CSMRI, the sparse regularization has been accomplished through a particular transform domain, such as the wavelet [10] and curvelet [48], or through some dictionary learning approaches [49–55]. The traditional CSMRI uses the fixed sparsifying transforms [56, 57]. Similarly, with recent development, many CNN-based deep learning methods [58–63] have also been evolved.

In this paper, the authors proposed a new efficient interpolated compressed sensing (EiCS) technique based on different radial undersampling patterns. The proposed sampling strategy reduces the undersampling ratio to even 3% by using a more practical undersampling approach. Secondly, the novel three-step interpolation approach ensures that each interpolated slice gets maximum samples from their respective target slice and the rest from their neighboring two slices, to have sufficient samples to be reconstructed as a sharper and improved image. The main contributions of this work are as follows:

- For the first time, iCS has been introduced with radial undersampling scheme
- Introducing an improved and novel three-step computational efficient interpolation approach
- Reduction in the scan time using the highest undersampling ratio
- Improving image quality (IQ) by introducing a new and practical undersampling strategy for interpolation
- Better results with even increased interslice gap datasets

The proposed algorithm is explained as Material and Methods in Section 2, results and discussion are presented in Section 3, Section 4 summarizes conclusion, and future work is presented in Section 5.

## 2. Materials and Methods

The proposed EiCS algorithm has three steps. Every step is elaborated in the following subsections.

### 2.1. Radial Undersampling Scheme

**Figure 3:**
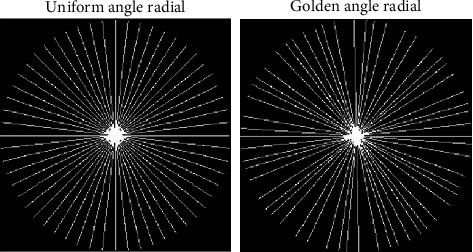
Two different radial sampling approaches used in this research.

The fully sampled *k*-space multislice MRI data greatly resembles 2D-VRDU and radial masks, as shown in Figure 2. The radial undersampling approach is more suitable as it is practical from the present hardware point of view compared to the 2D-VRDU undersampling schemes. Most importantly, the radial masks oversample the central region using overlapping spokes and thus detect and correct any movement in the *k*-space center for changes in between views. Thus, the motion artifacts in radial undersampling are averaged out because of the inherent oversampling of the *k*-space center. Therefore, the reconstructed images using the radial undersampling masks are less motion sensitive with higher SNR [18], compared to 1D- and 2D-VRDU schemes. When the radial data are acquired using the golden angle ratio [64], the *k*-space data are undersampled using high temporal incoherence [27]. Therefore, the sampling patterns explored in this research are uniform angle and golden angle radial as shown in Figure 3.

Unlike 1D- and 2D-VRDU, the radial samples are acquired on a polar grid. Therefore, the distance between the sampling points of the neighboring spokes is nonuniform. This distance is smaller in the *k*-space center and larger in the periphery. Thus, the radial readouts require regridding from the polar *k*-space data into pixel domain through a Density Compensation Function (DCF) and Nonuniform FFT (NUFFT) [19]. In uniform angle radial, all spokes are uniformly spaced while in golden angle radial every two spokes are spaced nonuniformly depending upon the golden angle ratio. The golden angle ratio (180°/((1+√5)/2) ≈ 111.246°) of radial sampling acquires the spokes such that they are self-interleaving and no spoke is acquired twice [64]. The number of spokes that is needed to meet the Nyquist sampling criteria is 402 uniformly spaced spokes, with 256 samples on each spoke for a 256 × 256 pixel image [22, 65]. Therefore, for acquiring 3%, 5%, 7%, and 9% of samples, we have to acquire 12, 20, 28, and 36 spokes, respectively.

**Figure 4:**
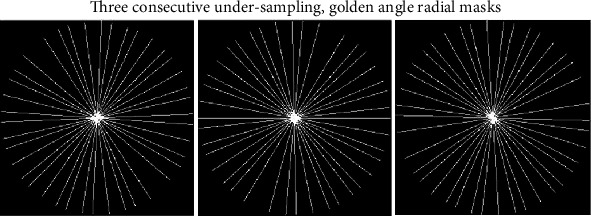
Three consecutive undersampling radial masks with nonoverlapping spokes.

The undersampling approach adopted with the proposed interpolation technique takes only 3%, radial samples, from every slice of the multislice MRI sequence. The proposed undersampling scheme is slice-wise uniform like CS, which means every slice is undersampled with the same undersampling ratio but using different nonoverlapping spokes. In the proposed EiCS technique, first, three different undersampled radial masks with the same sampling ratios are generated, as shown in Figure 4. These masks are then used to undersample three consecutive slices and repeated after every three slices for the whole multislice MRI sequence. Let *R*_1_, *R*_2_, and *R*_3_ be the three desired nonoverlapping undersampling radial masks. The three consecutive undersampling radial masks are such that they have the same number of spokes but have different sampling locations as shown in Figure 4.

Two fully sampled, original multislice MRI datasets of the knee are used for this research. But first, the multislice MRI datasets are undersampled into *k*-space data, and then, the proposed iCS technique is applied. For the undersampling of three consecutive slices *S*_i_, *S*_*i*+1_, and *S*_*i*+2_ first three downsampling NUFFT operators of the proposed sampling patterns are generated. The three downsampling NUFFT operators are termed as NUFFT_1_, NUFFT_2_, and NUFFT_3_, where each NUFFT operator is generated using its respective radial undersampling mask *R* and a DCF. To interpolate the *k*-space data from the non-Cartesian trajectories, the NUFFT by Fessler and Sutton [66] and the NUFFT wrapper by Lustig et al. [10] are implemented, which are available online [67, 68]. The NUFFT operators are then applied on three consecutive slices, resulting in an undersampled *k*-space slice sequence as represented in

(1) $$\begin{array}{c} U_{i}={\text{NUFFT}}_{1}*S_{i},\\ U_{i+1}={\text{NUFFT}}_{2}*S_{i+1},\\ U_{i+2}={\text{NUFFT}}_{3}*S_{i+2}, \end{array}$$

where *U*_*i*_, *U*_*i*+1_, and *U*_*i*+2_ represent the three consecutive undersampled slices. This undersampling step for three consecutive slices is repeated after every three slices for the whole multislice MRI dataset. Thus, the result will be an undersampled dataset in which every three consecutive slices have the same sampling patterns and undersampling ratios but different sampling locations because of using different nonoverlapping spokes, as represented in Figure 4. All the samples that are in different positions can be exploited for the proposed interpolation technique in the next step.

### 2.2. Proposed Efficient Interpolation Scheme

The proposed efficient interpolation scheme approximates the missing sampling points of each undersampled slice from their two neighboring slices. This approach works by considering any three consecutive downsampled slices, out of which the central one is termed as the target slice (*T* slice) which has to be interpolated from its left slice (*L* slice) and right slice (*R* slice). The proposed interpolation scheme has three steps. The first step is to find the set difference between the *L* and *T* slices as represented in

(2) $$\begin{array}{c} L_{T\text{new}}=L⊖T. \end{array}$$

The resultant set difference is called *L*_*T*new_, having the new information of the *L* slice with respect to the *T* slice, where the ⊖ sign represents the set difference operator. Secondly, the same step of *L* slice is repeated with the *R* slice, getting *R*_*T*new_, as shown in

(3) $$\begin{array}{c} R_{T\text{new}}=R⊖T, \end{array}$$

where *R*_*T*new_ contains the new sampling information in *R* slice with respect to the *T* slice. In the third and last step, the *T* slice samples are combined with the samples of *L*_*T*new_ and *R*_*T*new_ to get the interpolated *T* slice termed as *T*_int_ as represented in Equation (4), where the ⊕ sign is the set addition operator.

(4) $$\begin{array}{c} T_{\mathrm{int}}=L_{T\text{new}}⊕T⊕R_{T\text{new}}. \end{array}$$

**Figure 5:**
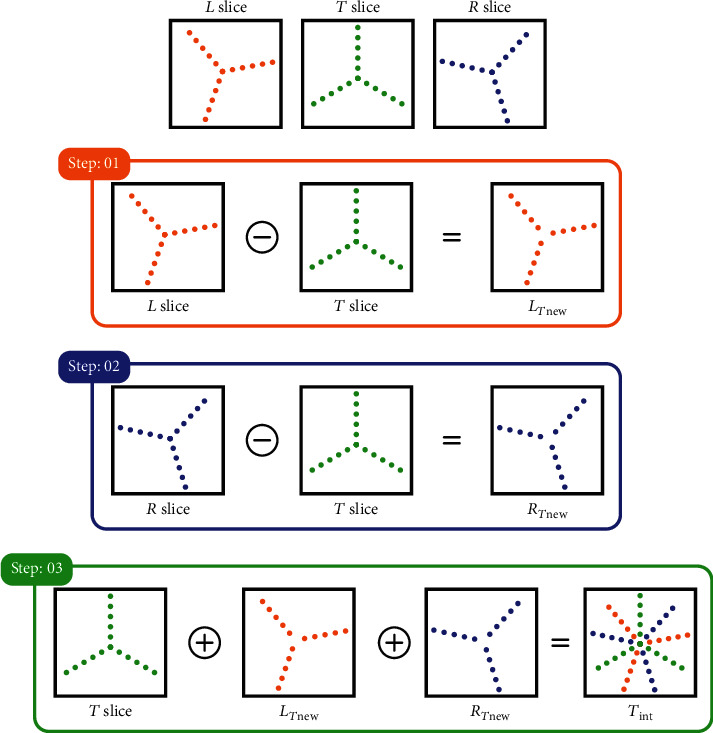
The proposed efficient interpolation scheme.

This three-step interpolation approach of EiCS is applied on each slice of the undersampled multislice MRI sequence, considering every slice as a *T* slice and its two neighboring as *L* and *R* slices, to acquire an interpolated slice *T*_int_. The three-step process of the proposed efficient interpolation scheme is represented in Figure 5.

### 2.3. CS Reconstruction

**Figure 6:**
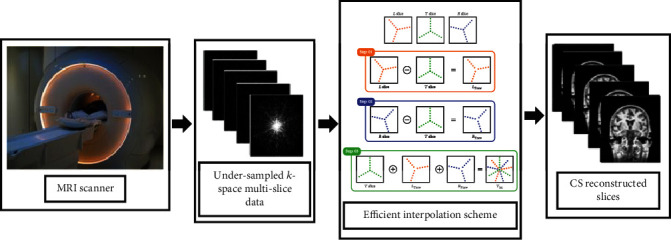
The proposed EiCS technique.

After the interpolation step, the interpolated multislice datasets have almost three times of the samples initially undersampled or acquired. The third and final step of EiCS is the CS reconstruction which gives the reconstructed images. The CS reconstruction technique used in this research is the nonlinear conjugate gradient (NCG) with *ℓ*_1_-norm and total variance (TV) [10] as represented in

(5) $$\begin{array}{c} \hat{x}=\arg\underset{x}{\min}{\left|\left|F_{u}x-y\right|\left|{}_{2}^{2}+\lambda_{1}\right|\left|\Psi x\right|\left|{}_{1}+\lambda_{2}\right|\left|x\right|\right|}_{\text{TV}}, \end{array}$$

where *y* is the *k*-space measurement, *F*_*u*_ is a downsampled Fourier operator, and Ψ is the wavelet operator. Thus, the cost function is minimized with the given constraints to reconstruct the image *x*. Similarly, *ℓ*_1_-norm is the objective function as represented in Equation (6), and minimizing ‖Ψ*x*‖_1_ promotes sparsity. Similarly, the constraint ‖*F*_*u*_*x* − *y*‖2/2 enforces data consistency, where *λ*_1_ and *λ*_2_ are the thresholding parameters for *ℓ*_1_ wavelet and TV penalty, respectively. TV is expressed discretely in Equation (7).

(6) $$\begin{array}{c} {\left\|x\right\|}_{1}=\underset{i}{\sum }\left|x_{i}\right|, \end{array}$$

(7) $$\begin{array}{c} {\left\|x\right\|}_{\text{TV}}=\underset{i,j}{\sum }\;\left[{\left(\nabla_{1}x_{ij}\right)}^{2}+{\left(\nabla_{2}x_{ij}\right)}^{2}\right], \end{array}$$

where ∇_1_ and ∇_2_ are the forward finite difference operators on the first and second coordinates. The complete EiCS technique is expressed in Figure 6.

## 3. Results and Discussion

The proposed EiCS technique is evaluated using two different knee datasets, taken from an online database, http://mridata.org. The knee datasets are fully sampled, acquired using a GE HD 3T scanner having 256 slices, number of channels: 8, 160 × 160 × 153.6 mm field of view, slice thickness 0.6 mm, matrix size: 320 × 320, TR/TE: 1150/25 msec, bandwidth 50 kHz, and flip angle 90°. The proposed EiCS algorithm is simulated using MATLAB 2016-a, with 16 GB RAM, 64-bit operating system, and 2.6 GHz Intel Core i7 processor.

### 3.1. Evaluation Criteria

To evaluate the quality of the reconstructed images, two approaches are used, full reference (FR) and nonreference (NR). In the FR approach, the quality of the reconstructed images is evaluated with respect to the original image where for the NR approach no original image is required. For the FR approach, five assessment parameters are used which are structural similarity index measurement (SSIM) [69], feature similarity index measurement (FSIM) [70], mean square error (MSE) [71], peak signal to noise ratio (PSNR) [71], and correlation (CORR) [72]. For the NR approach, two assessment parameters are used which are sharpness index (SI) [73] and perceptual image quality evaluator (PIQE) [74].

SSIM and FSIM give normalized mean values of structural similarity and feature similarity between the original and reconstructed images as represented in Equations (8) and (9).

(8) $$\begin{array}{c} \text{SSIM}\left(x,y\right)=\frac{\left(2\mu_{x}\mu_{y}+c_{1}\right)\left(2\sigma_{xy}+c_{2}\right)}{\left(\mu_{x}^{2}+\mu_{y}^{2}+c_{1}\right)\left(\sigma_{x}^{2}+\sigma_{y}^{2}+c_{2}\right)}, \end{array}$$

where *x* and *y* are the original and reconstructed images with size *m* × *n*. Similarly, *μ*_*x*_ and *μ*_*y*_ are the mean, *σ*_*x*_^2^ and *σ*_*y*_^2^ are the variances, and *σ*_*xy*_ is the covariance of *x* and *y*. Similarly, *c*_1_ = (*k*_1_*L*)^2^ and *c*_2_ = (*k*_2_*L*)^2^ are the variables used to stabilize the division, where *L* represents the dynamic range of the image and *k*_1_ and *k*_2_ are small constants.

(9) $$\begin{array}{c} \text{FSIM}\left(x,y\right)=\frac{\sum_{i,j}\left[S_{\text{PC}}.S_{G}\right].\left[\max\left({\text{PC}}_{x},{\text{PC}}_{y}\right)\right]}{\sum_{i,j}\max\left({\text{PC}}_{x},{\text{PC}}_{y}\right)}, \end{array}$$

where PC_*x*_ and PC_*y*_ are the phase congruency of original and reconstructed images and *S*_PC_ is the similarity measure for PC_*x*_ and PC_*y*_. Similarly, *S*_*G*_ is the similarity measure for gradient magnitude values for original and reconstructed images.

MSE is the most common FR estimator of image quality with values near to zero are better. The MSE between the original and reconstructed images can be calculated as in

(10) $$\begin{array}{c} \text{MSE}=\frac{1}{mn}\overset{m-1}{\underset{i=0}{\sum }}\;\overset{n-1}{\underset{j=0}{\sum }}{\left[x\left(i,j\right)-y\left(i,j\right)\right]}^{2} \end{array}$$

PSNR is the ratio between the maximum possible power of the original image with MSE, and because of the dynamic range of the signals, it is calculated as the logarithm term of the decibel scale [75] as given in

(11) $$\begin{array}{c} \text{PSNR}\left(\text{in }dB\right)=10\log_{10}\frac{{\left({\mathrm{MAX}}_{x}\right)}^{2}}{\text{MSE}} \end{array}$$

CORR between the original and reconstructed images is defined in Equation (12), having a normalized value and is better when close to one.

(12) $$\begin{array}{c} \text{CORR}=\frac{\sum_{i,j}\left\{\left[x\left(i,j\right)-\mu_{x}\right]\left[y\left(i,j\right)-\mu_{y}\right]\right\}}{\sqrt{\left\{\sum_{i,j}{\left[x\left(i,j\right)-\mu_{x}\right]}^{2}\right\}\left\{\sum_{i,j}{\left[y\left(i,j\right)-\mu_{y}\right]}^{2}\right\}}}. \end{array}$$

SI is the NR IQ assessment parameter and is derived from the intensity distribution in an image; its mathematical description is given in

(13) $$\begin{array}{c} \text{SI }\left(x\right)=-\log_{10}\left[\frac{\mu_{\text{TV}\left(x\right)}-\text{TV}\left(x\right)}{\sigma_{\text{TV}\left(x\right)}}\right], \end{array}$$

where *μ*_TV(*x*)_ and *σ*_TV(*x*)_^2^ are the mean and variance of TV(*x*). TV(*x*) is the total variance of the input image as shown in Equation (7).

PIQE is also a NR image quality score, as shown in Equation (14), lies in the range (0-100), and is inversely related to the perceptual quality of an image, which means the lower the value the higher the quality of the image.

(14) $$\begin{array}{c} \text{PIQE}=\frac{\left(\sum_{k=1}^{N_{\text{SA}}}D_{sk}\right)+C_{1}}{N_{\text{SA}}+C_{1}}, \end{array}$$

where *N*_SA_ indicated the number of spatially active blocks in a given image, *D*_*sk*_ is the amount of distortion in a given block, and *C*_1_ is a positive constant.

The proposed EiCS technique is evaluated using all the seven assessment parameters and compared with recent interpolation techniques [32, 38] and CS [10]. The proposed technique is also evaluated on whole datasets for different undersampling ratios and compared. Finally, the proposed technique is evaluated for three different interslice gaps.

### 3.2. Evaluation of the Proposed Undersampling Scheme

Like FiCS and CS, the proposed radial undersampling strategy of EiCS equally undersamples the *k*-space multislice MRI sequence but using a much lower undersampling ratio.  Table 1 shows a comparison of the proposed radial undersampling schemes with the 2D-VRDU undersampling scheme of FiCS [38], 1D-VRDU scheme of iCS [32], and CS [10]. The assessment has been performed using all the seven assessment parameters for three successive slices and averaged. It is clarified from the comparison of the different undersampling strategies in the table that the proposed radial undersampling scheme has more improved results, for both uniform angle and golden angle strategy, compared to 1D- and 2D-VRDU schemes. The radial sampling strategy has also an edge in that it is more practical from the current hardware point of view. In Table 1, the results of the interpolation approach of EiCS have not been included because in this section the proposed undersampling scheme of EiCS is evaluated. The interpolation technique of EiCS is evaluated and discussed in the next section.

### 3.3. Evaluation of the Proposed Efficient Interpolation Scheme

**Figure 7:**
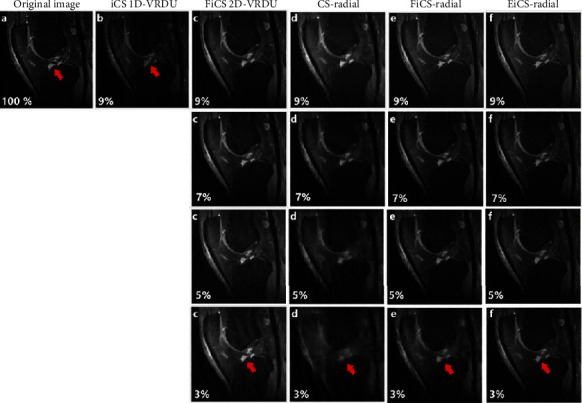
Comparison of (a) original image with (b–f) reconstructed images using different reconstruction strategies and sampling ratios. (b) iCS reconstructed image has sharper details but with loss of information. (c) FiCS 2D-VRDU has preserved the original information but has a blurring effect which becomes more prominent when the undersampling ratio is reduced. (d) CS reconstruction using the proposed radial undersampling also shows severe degradation when the sampling ratio reduces. (e) FiCS-radial and (f) EiCS-radial has improved results compared to CS-radial but the sharpness degrades for (e) FiCS with 3% samples while the proposed (f) EiCS has improved results with clear and sharp details as pointed by the red arrow.

The proposed three-step interpolation scheme of EiCS is evaluated by comparing its reconstructed images with that of iCS [32], FiCS [38], and CS [10].  Figure 7 shows a comparison of the original image with the reconstructed images using different interpolation techniques with different undersampling ratios. It is clear from the figure that although iCS shows visually improved results it represents information of the neighboring slices as iCS used a biased undersampling scheme [38]. FiCS using 2D-VRDU undersampling shows improved results and has no loss of information but their sampling pattern is nonrealistic with some blurring effect. CS reconstruction is performed using the proposed radial sampling pattern, but their images look even more blur with some streaking artifacts. The reconstructed images of the proposed radial sampling pattern show improved results for both FiCS and EiCS techniques. But EiCS, due to its three-slice interpolation approach, has better results compared to FiCS, using the same radial sampling strategy. This proves that the three-step EiCS technique is better compared to its preceding two-step FiCS technique. The edge information pointed by the red arrow in Figure 7 shows that although FiCS-radial has improved results, for 3% samples, it has a blurring effect, while EiCS has no blurring effect with sharper and clear details. Although FiCS 2D-VRDU also shows better results, its undersampling approach is nonrealistic with a bit blurred edges. In short, EiCS has got the benefits of all the other techniques as its reconstructed images have no blurring effect with sharper details and original information with a more realistic sampling approach using only 3% samples.

In the proposed EiCS technique, the acquired undersampled *T* slices when interpolated as *T*_int_ have 34% samples from *T* slices and 33% from each *L* and *R* slices. These percentages are calculated from the interpolated slices with reference to the original undersampled slices. In FiCS [38], every *T*_int_ slice has 60% samples from *T* slice and the rest 40% from its respective *L* slice. In FiCS, although a greater percentage of samples were taken from the original slices but because of their two-step interpolation approach, when the sampling ratio further reduces, the interpolated slices got insufficient samples to be reconstructed as a clear and sharper image. In iCS [25, 32], each interpolated slice has only 4% samples from its original undersampled slice and the remaining 96% from its corresponding neighboring slices. The reconstructed images of iCS show sharp details due to more samples in their interpolated slices but with the limitation that their resultant three consecutive reconstructed images show repeated information because of their biased undersampling strategy, as discussed in [38]. Although the 1D sampling scheme of iCS is also practical from the current hardware point of view, it has three times higher undersampling ratio, with a biased undersampling strategy.

Table 2 shows the total percentage of the original and interpolated samples for different reconstruction techniques. It is clear from the table that our proposed sampling strategy has the lowest percentage of samples from the neighboring slices and still has the highest percentage of interpolated samples, which give us the benefit that information content is original and the reconstructed images are sharper.

**Figure 8:**
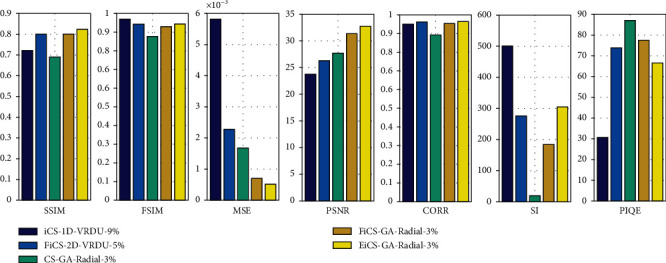
Comparison of the proposed EiCS averaged assessment parameters with iCS, FiCS, and CS. iCS 1D-VRDU has 9% and FiCS 2D-VRDU has 5% while CS-radial, FiCS-radial, and EiCS-radial have 3% samples. The proposed EiCS-radial technique outperforms all with even 3% average samples.

The proposed efficient interpolation scheme (EiCS) is also evaluated by comparing the seven assessment parameters of EiCS with iCS [32] and CS [10]. For more fair comparison, FiCS using the proposed radial sampling strategy is also performed. The proposed EiCS technique has not only improved performance with the same average undersampling ratio (5%) of FiCS but also outperforms with even a 3% sampling ratio as shown in Table 3. The assessment has been done on 3 consecutive slices and averaged. Table 3 represents a detailed evaluation where Figure 8 shows a brief summary of it.

The graphs of Figure 8 clearly show that FiCS-radial has improved performance with even 3% samples which proves that the radial undersampling strategy is better than 2D-VRDU. Secondly, the proposed EiCS-radial outperforms FiCS-radial which proves that the three-step interpolation technique of EiCS is better than the two-step approach of FiCS. EiCS-radial is also better than iCS 1D-VRDU with even one-third of the samples, but in three out of the seven assessment parameters (FSIM, SI, and PIQE), iCS looks better. The reason is that, firstly, iCS has 9% samples and, secondly, iCS has a biased sampling strategy, by taking 96% of samples from neighboring slices. Therefore, iCS shows better feature similarity, sharpness, and perceptual image quality but represents neighboring slice information. Thus, EiCS-radial beats all other techniques by taking only 3% samples.

**Figure 9:**
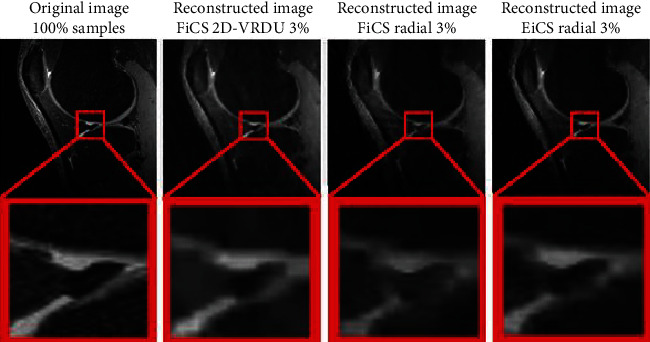
Comparison of the original image with reconstructed images using FiCS 2D-VRDU 3%, FiCS-radial 3%, and EiCS-radial 3%. It is clear from the comparison of the selected zoomed portions that the proposed EiCS technique outperforms FiCS by retaining sharper details.

Figure 9 represents a comparison of the original image with that of the reconstructed images using FiCS 2D-VRDU, FiCS-radial, and EiCS-radial with 3% samples by considering a zoomed edge. It is clear from the figure that EiCS-radial is better than both FiCS 2D-VRDU and FiCS-radial by showing clearer and sharper results.

### 3.4. Evaluation of EiCS

**Figure 10:**
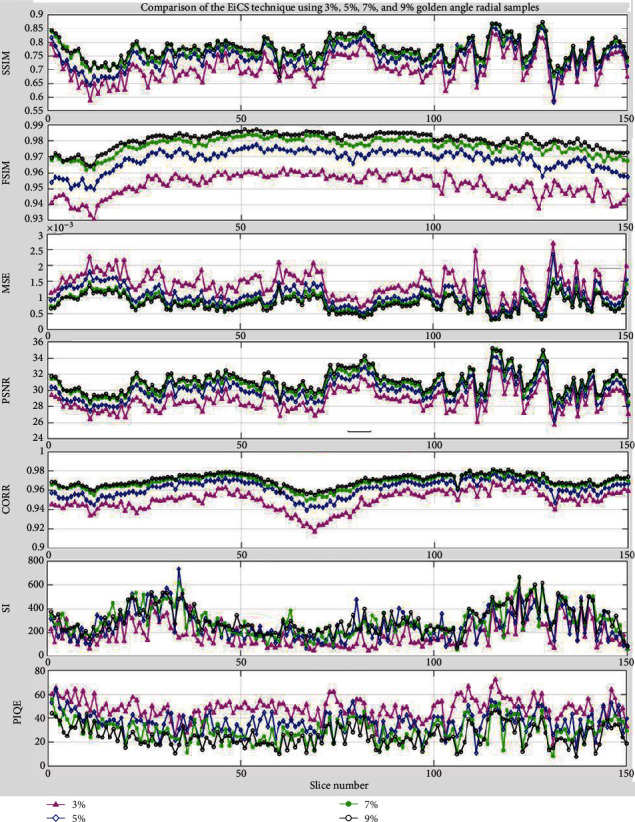
Evaluation of EiCS using the seven assessment parameters with GA-radial undersampling for 3%, 5%, 7%, and 9% samples. The evaluation has been done on central 150 slices of the knee dataset and compared slice-wise.

The detailed evaluation of the proposed EiCS technique is done on central 150 slices of the knee dataset (slices # 51 to 200) as shown in Figure 10. The evaluation is done using GA-radial undersampling scheme for all the seven assessment parameters. It is clear from the figure that when we increase the undersampling ratio the performance improves, but while increasing the sampling ratio from 7% to 9%, the total number of interpolated samples saturates and is oversufficient for CS reconstruction. Thus, as clear from the figure, when the sampling ratio increases from 7% to 9%, the proposed EiCS technique shows lesser improvement. This is because the three-step EiCS technique collects sufficient samples from reduced undersampling ratios that give improved results, with even 3% samples. Secondly, Figure 10 shows that EiCS has consistency in its results like FiCS where iCS shows inconsistent results as discussed in [38].

### 3.5. Evaluation of EiCS

**Figure 11:**
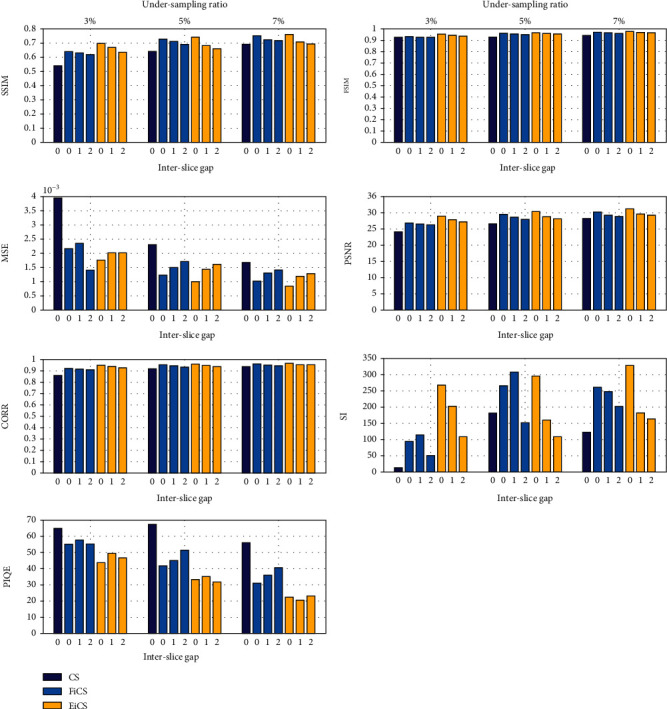
Comparison of FiCS and EiCS for different interslice gaps and undersampling ratios.

The proposed EiCS technique also outperforms for increased interslice gap datasets. The zero interslice gap means considering all the slices of the original dataset. One and two interslice gaps mean to skip one and two slices from consecutive slices, while taking two slices. Increasing the gap helps to further reduce the average undersampling ratio from 3% to 1.5% and 1%. Skipping one and two slices means that we are considering 128 and 85 slices from the 256-slice dataset.  Figure 11 shows the evaluation of CS, FiCS, and EiCS on radial sampling for 3%, 5%, and 7% undersampling ratios with zero, one, and two interslice gaps.

It is clear from the graphs of Figure 11 that both FiCS and EiCS have improved performance compared to CS for even increased interslice gaps. While comparing the performance of FiCS and EiCS, for higher interslice gaps, EiCS is better for lower undersampling ratios, but for 7% and higher ratios, FiCS is better on some assessment parameters. The reason is that for lower undersampling ratios, when the interslice gap is increased, EiCS, because of its three-slice approach, collects sufficient samples for improved reconstruction. Therefore, for higher undersampling ratios, when we increase the gap, FiCS performs better because of having sufficient samples using its two-slice approach, while for lower undersampling ratios, EiCS is better.

## 4. Conclusion

In this research, for the first time, interpolation has been proposed using radial undersampling schemes. The radial sampling pattern used with EiCS is more practical from the current hardware point of view compared to the 2D-VRDU sampling pattern adopted in FiCS. Secondly, the radial sampling strategy is also lesser motion sensitive compared to other sampling techniques. EiCS exploits the radial sampling pattern in its three-step interpolation process to get interpolated slices with the maximum number of samples from lower undersampling ratios, which ensures sharper reconstructed images compared to FiCS. The proposed EiCS technique not only preserves the original information in every slice but also shows sharper IQ with improved results both qualitatively and quantitatively. The improved interpolation technique adopted in EiCS is computationally efficient with only a set difference and addition operations like FiCS. Thus, the computational complexity of the proposed interpolation algorithm is *O*(*n*) like FiCS, compared to *O*(*n* log *n*) of iCS [32]. The key findings of this paper are as follows:

- Improved three-step interpolation scheme compared to its preceding two-step approach of FiCS
- Interpolation has never been explored using radial sampling schemes
- Computationally efficient like FiCS
- Reduced scan time, by further reducing the undersampling ratios compared to FiCS
- Improved IQ, by collecting maximum samples for sharper reconstruction
- The uniform undersampling pattern gives a consistent slice-wise image quality
- More realistic sampling scheme
- Improved result using seven different assessment parameters
- No blurring like FiCS
- No information loss and contrast change like iCS
- Best suitable for dynamic data as radial sampling can also handle motion artifacts

## 5. Future Work

The proposed EiCS technique can also be applied to dynamic MRI datasets to get even more benefits from radial sampling schemes. The proposed technique can be combined with the latest CS reconstruction strategies for more prominent results with reduced reconstruction time.

## Figures and Tables

**Table 1:** Comparison of the proposed radial undersampling schemes with 1D- and 2D-VRDU schemes, along with CS. The assessment has been done on 3 consecutive slices and averaged (slices 165-167 of the knee dataset).

Assessment parameter	1D-VRDU CS-9%	1D-VRDU iCS-9%	2D-VRDU CS-5%	2D-VRDU FiCS-5%	UA radial CS-5%	UA radial FiCS-5%	GA radial CS-5%	GA radial FiCS-5%
SSIM	0.5596	0.7226	0.7834	0.8008	0.7995	**0.8388**	0.7669	**0.8339**
FSIM	0.9387	0.9733	0.9713	0.9463	0.9201	**0.9593**	0.9150	**0.9516**
MSE	0.0398	0.0058	0.0056	0.0023	0.0008	**0.0004**	0.0010	**0.0005**
PSNR	14.002	23.779	22.636	26.367	30.738	**33.221**	29.797	**32.778**
CORR	0.9317	0.9522	0.9762	0.9647	0.9476	**0.9703**	0.9358	**0.9671**
SI	48.88	501.64	374.46	275.61	175.06	**267.63**	46.07	**276.44**
PIQE	65.791	30.915	62.121	73.850	72.577	**54.582**	79.574	**65.978**

**Table 2:** Comparison of %age number of samples acquired from the original slices for different interpolation techniques along with CS.

Reconstruction techniques	Samples taken from original slices (%)	Samples interpolated from neighboring slices (%)	Total samples for reconstruction with 9% average sampling
CS	100%	0%	9%
iCS	4%	96% from *L* slice	25%
FiCS	60%	40% from *L* slice	16%
EiCS	34%	33% from each *L* and *R* slice	26%

**Table 3:** Comparison of the proposed EiCS technique with CS, FiCS, and iCS for different undersampling ratios.

Average undersampling ratio	Interpolation technique	Undersampling technique	SSIM	FSIM	MSE	PSNR	CORR	SI	PIQE
9%	**iCS**	1D-VRDU	0.7226	0.9733	0.0058	23.779	0.9522	501.64	30.91

7%	**CS**	Uniform angle radial	0.8377	0.9464	0.00056	32.532	0.9653	303.48	68.65
		Golden angle radial	0.8322	0.9433	0.0006	32.174	0.963	220.62	76.48
	**FiCS**	Uniform angle radial	0.86	0.9705	0.00038	34.208	0.9764	449.19	54.5
		Golden angle radial	0.8608	0.9649	0.00039	34.057	0.9756	516.65	61.76
	**EiCS**	Uniform angle radial	**0.8559**	**0.9764**	**0.00034**	**34.658**	**0.9787**	**462.52**	**49.15**
		Golden angle radial	**0.8627**	**0.9733**	**0.00033**	**34.806**	**0.9795**	**442.74**	**50.82**

5%	**CS**	Uniform angle radial	0.7995	0.9201	0.00084	30.738	0.9476	175.06	72.57
		Golden angle radial	0.7669	0.915	0.00104	29.797	0.9358	46.07	79.57
	**FiCS**	Uniform angle radial	0.8388	0.9593	0.00047	33.221	0.9703	267.63	54.58
		Golden angle radial	0.8339	0.9516	0.00052	32.778	0.9671	276.44	65.97
	**EiCS**	Uniform angle radial	**0.8495**	**0.9681**	**0.00037**	**34.232**	**0.9765**	**476.98**	**45.33**
		Golden angle radial	**0.845**	**0.9673**	**0.0004**	**33.913**	**0.9748**	**338.01**	**50.94**

3%	**CS**	Uniform angle radial	0.7003	0.8768	0.00176	27.537	0.8887	20.607	80.9
		Golden angle radial	0.6922	0.8794	0.00171	27.665	0.895	19.44	87.34
	**FiCS**	Uniform angle radial	0.8164	0.9437	0.00063	32.003	0.9606	229.16	70.39
		Golden angle radial	0.8027	0.9318	0.00072	31.397	0.9546	183.73	77.5
	**EiCS**	Uniform angle radial	**0.8222**	**0.9512**	**0.00053**	**32.758**	**0.9671**	**251.05**	**56.84**
		Golden angle radial	**0.8235**	**0.9452**	**0.00053**	**32.684**	**0.9666**	**305.48**	**66.56**

## Data Availability

The proposed EiCS technique is evaluated using different knee datasets, taken from an online database, http://mridata.org. Similarly, to interpolate the *k*-space data from the non-Cartesian trajectories, the NUFFT by Fessler and Sutton [66] and the NUFFT wrapper by Lustig et al. [10] are implemented, which are available online [67, 68].
